# Solitary intracranial metastasis as the sole relapse site in gastric cancer patients after neoadjuvant therapy and surgery: a case series of 12 patients and clinical implications

**DOI:** 10.3389/fonc.2026.1838410

**Published:** 2026-05-29

**Authors:** Weijie Zhao, Guanglong Chen, Yijie Ma, Zhili Zhao, Baozhen Ma, Lingdi Zhao

**Affiliations:** 1 Department of General Surgery, the Affiliated Cancer Hospital of Zhengzhou University & Henan Cancer Hospital, Zhengzhou, Henan, China; 2 Department of Medical Oncology, the Affiliated Cancer Hospital of Zhengzhou University & Henan Cancer Hospital, Zhengzhou, Henan, China; 3 Department of Immunotherapy, the Affiliated Cancer Hospital of Zhengzhou University & Henan Cancer Hospital, Zhengzhou, Henan, China

**Keywords:** cardia cancer, gastric cancer, intracranial metastasis, isolated recurrence, lymph node metastasis, neoadjuvant therapy

## Abstract

**Background:**

Gastric cancer recurrence commonly involves the peritoneum, liver, and lungs, whereas isolated intracranial metastasis is rare, particularly in patients receiving neoadjuvant therapy and surgery. This study aimed to characterize a distinct subgroup of patients with isolated intracranial recurrence after neoadjuvant treatment and radical surgery.

**Methods:**

We retrospectively reviewed patients with gastric cancer who underwent neoadjuvant therapy followed by curative resection and subsequently developed isolated intracranial metastasis confirmed by whole−body imaging. Clinical and pathological features were systematically analyzed.

**Results:**

Twelve patients met the inclusion criteria. All patients were lymph node−positive (100%). The primary tumor was located at the cardia or esophagogastric junction in 11 patients (91.7%). Poorly differentiated histology was observed in eight patients (66.7%), while the remaining four (33.3%) had moderately differentiated tumors; no well−differentiated cases were identified. The median time from surgery to intracranial metastasis was 15.3 months (95% confidence interval: 9.0–21.6 months).

**Conclusions:**

Isolated intracranial metastasis after neoadjuvant therapy for gastric cancer is associated with a distinct clinical profile: cardia/esophagogastric junction location, positive lymph node status, and poorly differentiated histology. Recognition of this high−risk profile may facilitate early diagnosis and inform surveillance strategies in this patient population.

## Introduction

Gastric cancer is one of the most common malignancies worldwide, and its high recurrence rate is a major cause of mortality (1, 2). For locally advanced gastric cancer, the current standard treatment strategy primarily includes perioperative chemotherapy or chemotherapy combined with immunotherapy, followed by radical surgical resection (3–5). Although this comprehensive treatment model has significantly improved overall patient survival, a considerable proportion of patients still experience recurrence after treatment. The classic patterns of gastric cancer recurrence mainly include local recurrence, peritoneal dissemination, liver metastasis, and distant lymph node metastasis (6). In contrast, central nervous system metastasis in gastric cancer is extremely rare, with previous literature reporting an incidence of less than 1% among all gastric cancer patients (7). It is most commonly observed in patients with advanced-stage disease involving multiple organ metastases and is associated with an extremely poor prognosis (7, 8).

In recent years, with the continuous optimization of neoadjuvant treatment regimens - particularly the widespread use of immune checkpoint inhibitors into the neoadjuvant setting - the therapeutic outcomes for gastric cancer have further improved (4, 9). However, advances in systemic therapy have also given rise to a potential clinical concern: the central nervous system may serve as a “sanctuary” for tumor cells. Due to the presence of the blood–brain barrier, the concentrations of most chemotherapeutic agents and some targeted drugs in the cerebrospinal fluid are significantly lower than those in the peripheral blood. This pharmacological characteristic renders the intracranial space a “safe haven” for minimal residual disease, potentially leading to an evolution in recurrence patterns (10).

Recently, during clinical follow-up, we have identified a cohort of postoperative gastric cancer patients sharing distinct clinical characteristics. These patients all received neoadjuvant therapy and subsequently underwent successful R0 radical resection. During postoperative surveillance, although no evidence of liver, lung, or peritoneal recurrence was detected on thoracoabdominal imaging, intracranial metastases were incidentally discovered—either due to the emergence of neurological symptoms or through routine follow-up examinations. Moreover, whole-body PET-CT or contrast-enhanced CT confirmed that the brain was the sole site of recurrence. This pattern of “brain-only recurrence” markedly deviates from the typical recurrence patterns observed in gastric cancer, suggesting the possible existence of a distinct biological subtype or a therapy-induced selection effect.

To date, case series studies focusing on isolated brain recurrence following neoadjuvant therapy in gastric cancer remain extremely limited. The associated clinicopathological characteristics, risk factors, molecular mechanisms, and optimal follow-up management strategies have yet to be clearly defined. In view of this, the present study retrospectively analyzed 12 gastric cancer patients who met the aforementioned criteria and were treated at our center in recent years. Their clinicopathological features, treatment courses, recurrence patterns, and survival outcomes were systematically reviewed. This analysis aims to enhance clinicians’ awareness of this rare recurrence pattern, explore its potential implications for postoperative surveillance strategies, and provide preliminary clinical insights to inform further investigation into the underlying molecular mechanisms.

## Materials and methods

### Patient population

This retrospective investigation was carried out at Henan Provincial Cancer Hospital. The study was performed in full compliance with the ethical guidelines set forth in the Declaration of Helsinki, and the research protocol was formally reviewed and approved by the Medical Ethics Committee of Henan Provincial Cancer Hospital (Approval No. 2025-279). As the study is retrospective in nature, the ethics committee granted a waiver of informed consent from the participating patients.

### Inclusion and exclusion criteria

The study’s inclusion criteria were defined as follows: 1) Histologically confirmed adenocarcinoma originating in the stomach or gastroesophageal junction (G/GEJ); 2) Initial clinical staging indicative of locally advanced disease (T3–T4 and/or node-positive, N+) with no evidence of distant metastases, as determined through contrast-enhanced CT imaging of the chest, abdomen, and pelvis (using iopromide, 1-mm slices) and/or endoscopic ultrasound; 3) Recommendation for neoadjuvant treatment established by a multidisciplinary tumor board specializing in G/GEJ cancers; 4) Completion of either neoadjuvant chemotherapy alone or in combination with immunotherapy; 5) Achievement of curative-intent surgery with microscopically margin-negative (R0) resection; 6) Diagnosis of isolated intracranial metastasis (affecting the brain parenchyma or meninges) during postoperative surveillance, with whole-body contrast-enhanced CT or PET-CT confirming the absence of extracranial disease.

The exclusion criteria for this study were defined as follows: 1) Evidence of distant metastasis at initial diagnosis, for which conversion therapy was administered; 2) A prior history of other primary malignancies before the initiation of neoadjuvant therapy; 3) Patients who, during follow-up, remained disease-free, experienced non-intracranial recurrence or metastasis, or presented with synchronous intracranial and extracranial metastases; 4) Incomplete follow-up data or loss to follow-up.

### Statistics

SPSS version 22.0 (IBM Corp., Armonk, NY, USA) was used for all statistical analyses. Continuous variables (age, days from diagnosis to surgery, number of neoadjuvant therapy cycles, number of lymph nodes detected) were expressed as median and range, CEA, CA199, and CA724 levels at initial diagnosis and preoperation were expressed mean ± standard deviation. Disease-free survival (DFS) was defined as the time interval from the date of surgery to the first detection of intracranial metastasis; Overall survival (OS) was defined as the interval from the diagnosis of gastric cancer to patient death or the last follow-up.

## Results

### Patients’ characteristics

**Figure 1:**
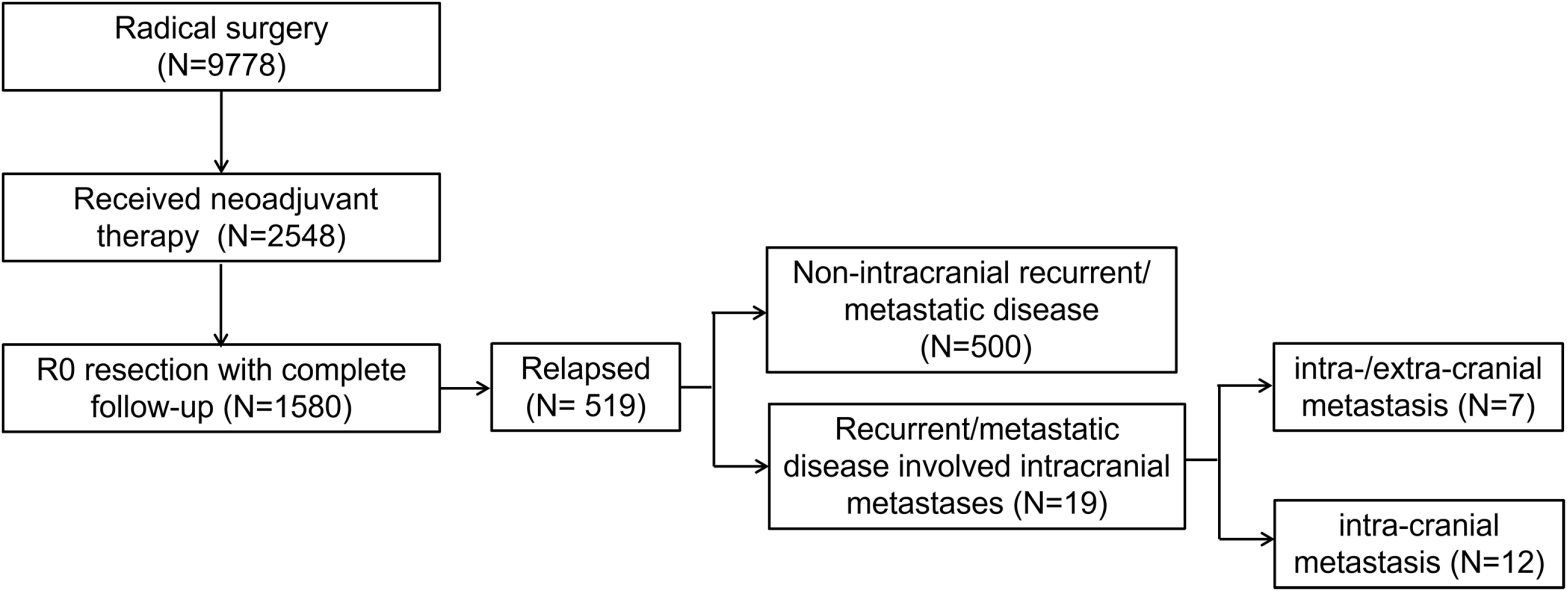
Screening flowchart of enrolled patients. Among those who underwent radical resection, patients receiving neoadjuvant therapy, achieving R0 resection, and having complete follow-up data were selected. Among patients who developed recurrence or metastasis during follow-up, those with brain‑only metastasis (n = 12) were finally included.

From January 2019 to December 2024, a total of 9778 patients at Henan Provincial Cancer Hospital underwent radical surgery for gastric cancer, of which 2,548 received neoadjuvant therapy prior to surgery. Among 2,548 patients who underwent surgery following neoadjuvant therapy, 1,580 with complete follow-up data remained after excluding those lost to follow-up or with R1 resection. Of these, 519 patients developed recurrence or metastasis during postoperative follow-up, including 19 patients with intracranial metastasis. Among the 19 patients with brain metastasis, six had concurrent extracranial lesions at the time of brain metastasis diagnosis, and one patient developed extracranial metastasis four months after the detection of brain metastasis. A flowchart detailing the patient screening, selection, and inclusion process is presented in **Figure 1**. A total of 12 patients presented with isolated intracranial metastasis throughout the disease course. **Table 1** presents the clinical characteristics of the 12 enrolled patients.

**Table 1:** Baseline characteristics of the patients enrolled.

Characteristics	Patient selection flowchart
Age (years)	
median (range)	61.5 (46-76)
Sex	
male	10 (83.3%)
Location primary (n%)	
G	1 (8.3%)
GEJ	11 (91.7%)
Differentiation (n%)	
Moderately	4 (33.3%)
Poorly	8 (66.7%)
Clinical T (n%)	
T3	5 (41.7%)
T4	7 (58.3%)
Clinical N (n%)	
N positive	12 (100%)
No. of nodes detected	
median (range)	24 (14-59)
Neoadjuvant regimen (n%)	
Chemotherapy	7 (58.3%)
Chemoimmunotherapy	5 (41.7%)
No. of perioperative cycles	
median (range)	6 (4-8)

### Efficacy of neoadjuvant therapy

Among the 12 patients, 2 had evaluable lesions according to the RECIST criteria and achieved a partial response (PR) following neoadjuvant therapy. The remaining 10 patients had no clinically evaluable lesions, with CT imaging indicating local disease improvement. No patients exhibited disease progression at the time of clinical efficacy assessment, with a disease control rate of 100%.

Pathological evaluation of tumor regression following surgery was performed in accordance with the Ryan criteria. Among the 12 patients, 3 exhibited a tumor regression grade (TRG) of 0, 2 had TRG 1, 6 had TRG 2, and 1 had TRG 3. A pathological complete response (pCR) was achieved in 3 out of 12 patients (25%), while a major pathological response (MPR) was observed in 5 out of 12 patients (41.7%).

### Adjuvant therapy after surgery

All patients received postoperative adjuvant therapy. Among them, two discontinued treatment after one cycle due to poor tolerance and subsequently underwent regular observation. The remaining 10 patients completed the planned 6 to 8 cycles of perioperative treatment. The perioperative treatment details for the 12 patients are shown in **Table 2**.

**Table 2:** Perioperative treatment of 12 patients involved.

No. of patient	Neoadjuvant regimen	No. of neoadjuvant	Adjuvant regimen	No. of adjuvant
1	SOX	4	SOX	2
2	FLOT plus toripalimab	3	S-1 monotherapy	1
3	SOX plus sintilimab	4	SOX plus sintilimab	4
4	SOX plus sintilimab	3	SOX	5
5	SOX	3	SOX	3
6	SOX	3	SOX	4
7	SOX	3	SOX	3
8	SOX	3	SOX	5
9	mFolfox6 plus camrelizumab	4	mFolfox6 plus camrelizumab	2
10	SOX plus sintilimab	3	SOX plus sintilimab	1
11	FLOT	4	FLOT	4
12	SOX	3	SOX	4

### Treatment after intracranial metastasis

As of February 28, 2026, the median follow-up time was 35.4 (IQR 19.7-51.4) months, during which 6 patients died. After the development of intracranial metastasis, two patients received symptomatic treatment only, one patient underwent surgical resection followed by regular follow-up, and another patient received only stereotactic radiosurgery (SRS) for the brain metastases followed by regular follow-up due to immune thrombocytopenia. The treatments for these 12 patients after intracranial metastasis are detailed in **Table 3**.

**Table 3:** Treatment course after intracranial metastasis.

No. of patient	Treatment course	Status
1	SRS, followed by nab-paclitaxel, oxaliplatin, and apatinib	alive
2	SRS, followed by apatinib plus cadonilimab	alive
3	Supportive care	dead
4	Whole-brain radiotherapy, sintilimab immunotherapy, progression of an isolated intracranial metastatic lesion and surgical resection	alive
5	SRS, followed by S1 plus apatinib	dead
6	surgical resection	dead
7	SRS, followed by fluorouracil plus cisplatin	dead
8	SRS	alive
9	Supportive care	dead
10	Surgical resection followed by SRS; S-1 plus sintilimab consolidation therapy	alive
11	SRS, XELOX plus sintilimab and trastuzumab for 5 cycles, followed by capecitabine, sintilimab, and trastuzumab therapy	alive
12	SRS, SOX plus sintilimab for 3 cycles, followed by sintilimab plus apatinib	dead

### Survival

**Figure 2:**
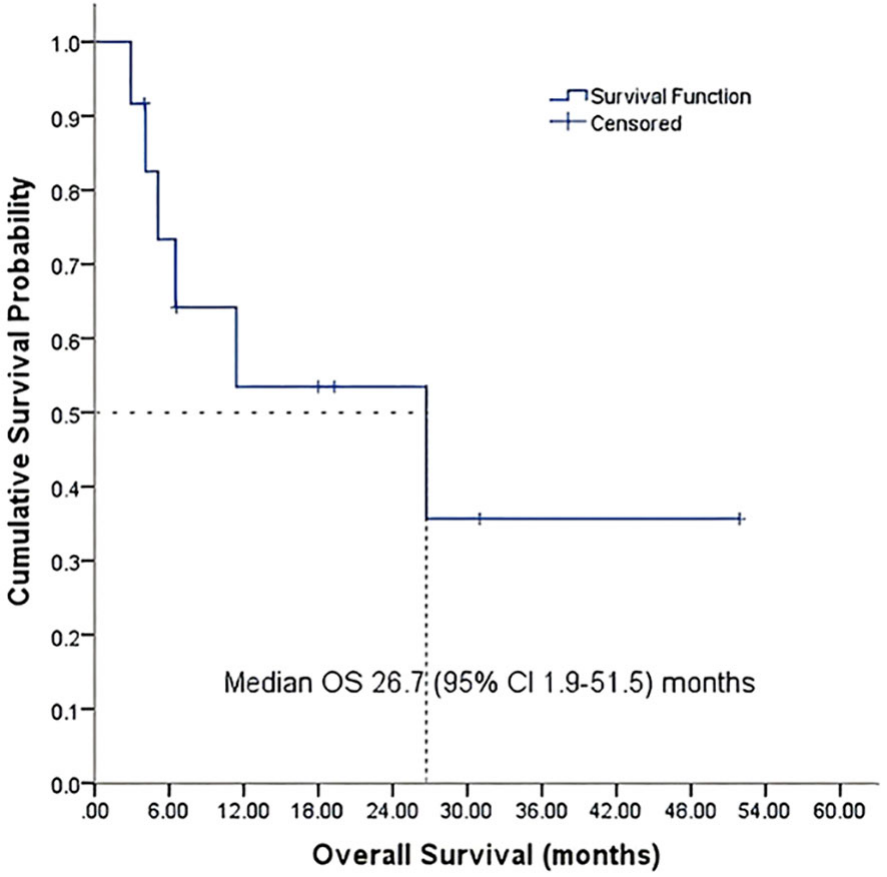
Kaplan-Meier survival curve illustrating cumulative survival probability over time (months) for patients with brain‑only metastasis. The median overall survival was 26.7 months (95% confidence interval: 1.9-51.5 months). Censored data points are indicated along the survival function curve.

During postoperative follow-up, all 12 patients developed intracranial metastasis, with a median time to intracranial metastasis of 15.3 months (95% confidence interval: 9.0–21.6 months). The median duration of post−brain−metastasis imaging follow−up among survivors was 9.0 months (range: 2.9–51.9 months). During this follow−up period, whole-body enhanced CT was typically performed at about the intervals of 2–6 months, none of the 12 patients developed detectable extracranial metastases. Six patients died. As of the last follow-up, the median overall survival after intracranial metastasis was 26.7 (95% CI 1.9-51.5) months. **Figure 2** was the OS curve and the OS was calculated as the time interval from intracranial metastasis to patient death or last follow-up.

## Discussion

This study systematically described the rare clinical phenomenon of isolated intracranial metastasis following neoadjuvant therapy and surgery in 12 patients with gastric cancer, and for the first time, delineated a patient profile for this group based on clinicopathological characteristics. The results reveal that these 12 patients share highly consistent clinical features: all were lymph node−positive; the majority (11/12, 91.7%) had primary tumors located in the GEJ; histological grading was predominantly poorly differentiated (8/12, 66.7%), with the remainder being moderately differentiated, and no well−differentiated cases were observed. This combination of features is uncommon in the general gastric cancer population but is highly enriched in this case series of isolated brain metastases, suggesting that it may constitute a distinct high−risk subtype for recurrence. The following discussion focuses on three key clinical factors - primary tumor site, lymph node status, and histological grade - to construct a comprehensive high−risk profile, along with its clinical implications and the limitations of the study.

In this case series, EGJ cancers accounted for 91.7% (11/12) of cases, a proportion far higher than their distribution in the general gastric cancer population. This marked enrichment suggests that the primary tumor site may be an important factor in predicting the risk of brain metastasis from gastric cancer. Adenocarcinoma of the GEJ exhibits distinct biological behavior that differentiates it from true esophageal cancer and distal gastric cancer, and it is widely recognized as an independent clinicopathological entity. Compared with distal gastric cancer, cardia/GEJ cancer demonstrates greater invasiveness and a higher propensity for lymph node metastasis. More importantly, the anatomical characteristics of this region determine its unique metastatic pathways. Classical theories of tumor metastasis propose that hematogenous spread typically follows the pathway of “veins → right heart → lungs → left heart → systemic circulation,” making pulmonary metastasis the initial manifestation of blood-borne dissemination. However, the cases in this series presented with “isolated intracranial recurrence” without pulmonary metastases, suggesting the existence of a “shortcut” that bypasses the lungs. This phenomenon can be reasonably explained by the Batson venous plexus hypothesis. The Batson venous plexus, also known as the vertebral venous plexus, is a valveless venous system extensively interconnected with veins of the thoracic, abdominal, and pelvic cavities. Under conditions of increased intra-abdominal pressure—such as coughing, sneezing, or straining during defecation—retrograde blood flow can occur, allowing tumor cells to directly enter the vertebral venous system and subsequently travel retrogradely to the intracranial venous sinuses and brain tissue without passing through the pulmonary capillary bed (11, 12). The venous drainage of the cardia/GEJ region is intimately associated with this system; tumor cells invading this area could enter the vertebral venous plexus via anastomotic channels and metastasize to the brain, potentially without concomitant pulmonary metastasis (13). Additionally, tumor cells may metastasize to the central nervous system via meningeal pathways. Studies suggest that tumor cells can spread hematogenously to the Batson venous plexus of the spinal cord and subsequently travel through the veins or the perivenous spaces of the venous plexus into the dural or arachnoid sleeves of nerve roots, ultimately reaching the subarachnoid space (14). This pathway similarly bypasses the lungs and may explain why these patients developed brain metastases without other visceral metastases.

In this case series, all 12 patients were lymph node−positive (100%), a feature that was highly consistent across all included cases. Lymph node positivity is not only a traditional marker of poor prognosis but, in the context of this study, more importantly suggests that the tumor cells have acquired the ability for systemic dissemination, laying the foundation for subsequent isolated brain recurrence. During tumor progression, regional lymph node metastasis is regarded as a critical juncture in the transition from “localized disease” to “systemic disease”. For tumor cells to complete the entire process of detaching from the primary lesion, invading lymphatic vessels, and surviving and proliferating within lymph nodes, they must acquire a series of molecular capabilities, including epithelial-mesenchymal transition (EMT), extracellular matrix degradation, chemokine receptor expression, angiogenic capacity, and immune evasion (15, 16). These very capabilities are also the core functions required for tumor cells to achieve distant organ metastasis. Therefore, lymph node-positive status itself may serve as a surrogate marker of metastatic potential. Lymph node positivity often indicates that tumor cells have already breached local anatomical barriers and entered the systemic circulation or lymphatic circulation, forming systemic minimal residual disease that is undetectable by imaging. Following neoadjuvant therapy, tumor cells in the peripheral or systemic circulation may be eliminated due to exposure to antineoplastic agent. However, cells that have escaped into the central nervous system via special routes such as the Batson venous plexus, or drug−resistant clones with stem-like properties within the primary tumor, may “survive” under the selective pressure of neoadjuvant therapy (17). Recent studies have revealed a close association between lymph node metastasis and cancer stem cells. Cancer stem cells possess self-renewal capacity, multilineage differentiation potential, and enhanced drug resistance, making them key drivers of tumor recurrence and metastasis (18). Lymph node metastases often harbor subpopulations of cells with stem-like characteristics, which, after detaching from the primary lesion, are more likely to colonize and grow in distant organs, including the brain.

In this case series, poorly differentiated adenocarcinoma accounted for two-thirds of cases (8/12), moderately differentiated for one-third (4/12), and no well-differentiated cases were observed. This distribution pattern stands in stark contrast to the proportion of poorly differentiated cases in the general gastric cancer population, suggesting that a poorly differentiated status may be a critical biological basis for isolated brain metastasis from gastric cancer. Poorly differentiated tumors typically exhibit pronounced EMT characteristics. EMT is a process by which tumor cells lose epithelial polarity and acquire a mesenchymal phenotype, accompanied by a series of molecular changes including downregulation of E-cadherin, upregulation of N-cadherin, and increased expression of vimentin (19). This transformation enables tumor cells to detach from the primary lesion, enhance migratory capacity, and resist apoptosis, thereby establishing the molecular foundation for subsequent brain metastasis. There is a close association between poorly differentiated tumors and cancer stem cells. Poorly differentiated tumors generally harbor a greater enrichment of cells with stem-like characteristics, which possess self-renewal capacity, phenotypic plasticity, and chemoresistance (18). In the context of this study, these stem-like tumor cells may “survive” neoadjuvant therapy, disseminate via specialized routes to the intracranial compartment, and progressively grow to form metastatic lesions within the brain tissue. The brain tissue microenvironment is fundamentally distinct from that of the gastric microenvironment, featuring differences in energy sources, the presence of the blood-brain barrier, and unique stromal cell populations. Due to their high degree of phenotypic plasticity, poorly differentiated tumor cells are capable of adapting to the brain microenvironment through mechanisms such as metabolic reprogramming, microenvironmental mimicry, and interaction with glial cells (20–22). This “plasticity-adaptability” characteristic enables poorly differentiated tumor cells to overcome diverse microenvironmental barriers and ultimately “take root” within the brain tissue.

Integrating the three dimensions above, this case series delineates a “high-risk profile” for isolated intracranial recurrence after neoadjuvant therapy for gastric cancer: primary tumor located in the cardia/esophagogastric junction + clinical lymph node positivity + poorly differentiated histologic grade. Based on this high-risk profile, we propose the following clinical management recommendations: (1) Identification of high-risk individuals: Patients with gastric cancer who simultaneously meet all three criteria above should be considered at high risk for isolated intracranial recurrence, and clinicians should maintain a high index of suspicion. (2) Optimization of follow-up strategies: For high-risk individuals, enhanced central nervous system surveillance should be considered. When patients present with neurological symptoms such as headache, vomiting, or seizures, contrast-enhanced cranial MRI should be performed immediately. Where resources allow, the benefit-cost ratio of routine postoperative surveillance cranial MRI may be explored. (3) Adjustment of treatment strategies: For high-risk individuals, central nervous system prophylaxis should be considered during adjuvant therapy. Priority should be given to agents with favorable blood-brain barrier penetration; for patients with HER2 positivity, targeted therapies capable of crossing the blood-brain barrier may be considered; for those sensitive to immunotherapy, the potential role of immune checkpoint inhibitors in brain metastasis warrants attention (23, 24).

We further explored whether the type of neoadjuvant systemic therapy influenced the timing or pattern of isolated brain recurrence. Among the 12 patients, 7 received chemotherapy alone and 5 received chemoimmunotherapy. However, no apparent differences were observed between the two subgroups in terms of the interval from primary surgery to brain recurrence. Nevertheless, given the small sample size in each subgroup, these comparisons are underpowered, and definitive conclusions cannot be drawn. The absence of observable differences should be interpreted with caution, and larger studies are warranted to assess whether the addition of immunotherapy alters the natural history of CNS metastasis in this high-risk population.

Admittedly, this study has the following limitations: First, it is a single-center, retrospective study with a small sample size (12 cases), which may introduce selection bias, and the resulting survival data (median OS: 26.7 months; 95% CI: 1.9–51.5 months) are therefore preliminary and must be interpreted with caution. Consequently, the generalizability of the findings should be approached with caution. Second, paired molecular biological analysis of primary tumors and brain metastases is lacking, precluding validation of the key molecular mechanisms driving brain metastasis at the genomic/transcriptomic level. Third, due to the difficulty in obtaining brain metastasis tissue, only a subset of patients had surgical specimens of brain metastases, limiting in-depth investigation of the metastatic microenvironment. Fourth, follow-up durations vary, and long-term survival data for some patients remain incomplete. Future multicenter prospective studies, large-scale molecular analyses, and animal model validations are needed to further elucidate the mechanisms underlying isolated brain metastasis from gastric cancer and to develop prevention and treatment strategies.

## Data Availability

The raw data supporting the conclusions of this article will be made available by the authors, without undue reservation.
